# Retracted: Possible Role of Interaction between PPAR*α* and Cyclophilin D in Cardioprotection of AMPK against* In Vivo* Ischemia-Reperfusion in Rats

**DOI:** 10.1155/2019/9760941

**Published:** 2019-07-15

**Authors:** PPAR Research

*PPAR Research* has retracted the article titled “ ” [1] due to errors found in the scientific content of the article. We consulted our Editorial Board who believed that myocardial ischemia-reperfusion (IR) for only 24 hours should not induce an increase in the heart weight-to-body weight ratio because cardiac hypertrophy takes 1-2 weeks, though the authors argued that the increase was due to an inflammatory response, which disappears after 2-3 days. The board queried what is meant when a 100% infarct area/total area ratio is reported for IR hearts, when the tetrazolium chloride (TTC) staining in Figure 1 shows a small area of infarct: the authors explained that the infarct size was normalized to the IR group as 100%. The TTC images are of low technical quality: some hearts have 8 slices and others have 10, making the results difficult to analyze. The infarcts in all groups are minor and TTC staining is minimal; many of the hearts have little ischemic injury and the board found the normalization misleading. Although the authors provided the underlying data and images, the board recommended retraction. The authors disagree with this decision.

For the Western blot results, the board argued that PPARalpha antibodies are difficult to use and robust results are surprising; the authors said they tested the specificity. The interaction of a cytosolic protein cyclophilin D with PPARalpha, a nuclear protein, is possible but does not make sense for the proposed mechanism. The identity of the band said to be PPARalpha is questionable: the phosphorylated PPARalpha is from a different blot (and thus a different sample) than the total PPARalpha and the GAPDH control. The AMPK blots have the same problem. Some of the images do not correspond to the underlying images.

The quantification of the co-immunoprecipitation (Co-IP) is questionable: the IgG lane is spliced into the image. As with the Western blots, using the PPARalpha antibody in this immunoprecipitation should have been quite difficult. The authors said Co-IP was performed using a “reverse approach”: IP with PPARalpha antibody followed by immunoblotting with CyP-D antibody and vice versa; IP with CyP-D antibody followed by immunoblotting with PPARalpha antibody.

The echocardiographic function data provided is incomplete: many parameters are shown in some mice, but not others. Ejection fraction values are given, but the volume measures needed to calculate this are not. There are several errors in the standard error calculations, because the “n” includes animals that were not assessed for the given parameters.

Finally, the board found it surprising that calcium-induced mitochondrial swelling was largely blocked in the drug-treated group.

## References

[1] Barreto-Torres G., Javadov S. (2016). Possible role of interaction between PPAR*α* and cyclophilin D in cardioprotection of AMPK against* in vivo* ischemia-reperfusion in rats. *PPAR Research*.

